# Biogeography of Soil Bacterial Networks along a Gradient of Cropping Intensity

**DOI:** 10.1038/s41598-019-40422-y

**Published:** 2019-03-07

**Authors:** Battle Karimi, Samuel Dequiedt, Sébastien Terrat, Claudy Jolivet, Dominique Arrouays, Patrick Wincker, Corinne Cruaud, Antonio Bispo, Nicolas Chemidlin Prévost-Bouré, Lionel Ranjard

**Affiliations:** 1 Agroécologie, AgroSup Dijon, INRA, Université Bourgogne Franche-Comté, F- 21000 Dijon, France; 2 INRA, US 1106, Unité INFOSOL, 45075 Orléans, France; 3 CEA/Institut de Génomique/Genoscope, 91057 Evry cedex, France; 4 ADEME, Service Agriculture et Forêt, 49000 Angers, France

## Abstract

Although land use drives soil bacterial diversity and community structure, little information about the bacterial interaction networks is available. Here, we investigated bacterial co-occurrence networks in soils under different types of land use (forests, grasslands, crops and vineyards) by sampling 1798 sites in the French Soil Quality Monitoring Network covering all of France. An increase in bacterial richness was observed from forests to vineyards, whereas network complexity respectively decreased from 16,430 links to 2,046. However, the ratio of positive to negative links within the bacterial networks ranged from 2.9 in forests to 5.5 in vineyards. Networks structure was centered on the most connected genera (called hub), which belonged to *Bacteroidetes* in forest and grassland soils, but to *Actinobacteria* in vineyard soils. Overall, our study revealed that soil perturbation due to intensive cropping reduces strongly the complexity of bacterial network although the richness is increased. Moreover, the hub genera within the bacterial community shifted from copiotrophic taxa in forest soils to more oligotrophic taxa in agricultural soils.

## Introduction

The response of soil bacterial communities to different environmental perturbations has been intensively investigated during the last decade. Studies have highlighted the significant role of soil characteristics^1–3^, plant communities, climate^2^ and land use^4^ as drivers of the abundance, diversity and structure of soil microbial communities. However, the influence of these drivers on the number and intensity of interactions occurring between community members remains little known, partly because microbes, and most of their interactions, cannot be directly observed or measured^5^. Biological relationships or interactions are essential to ecosystem functions as previously demonstrated and discussed with regard to macroorganisms, as exemplified by plant-pollinators systems^6,7^, mycorhizal interactions^8^ and the regulation of biotic invasions^9^. Recently, a new metric has been developed to determine and assess the relationships occurring in microbial communities. This approach, based on evaluation of the co-abundance between taxa, highlights the positive and negative biological relationships, also known respectively as co-occurrence and co-exclusion^10^. Positive relationships in microbial communities can result from cooperative or co-dependence interactions, *e.g*. the facilitation of organic matter degradation, or the similarity of ecological niches between microorganisms. Negative relationships, in contrast, can be attributed to inhibitive or antagonistic interactions such as antibiosis or competition for resources^11^. Thus, the co-occurrence approach contributes to a comprehensive evaluation of putative microbial relationships, independently of our technical capacity to observe and identify them^10^.

Analyzing the microbial co-occurrence network in soil could thus provide a promising way to improve our understanding of soil microbial community regulation, functioning and stability^10,12^. However, the relationships between the microbial interaction network and biological function have not been clearly demonstrated in complex ecosystems. The only examples to date have been obtained on simplified microbial ecosystems such as biofilms, where the co-occurrence and interaction of different bacterial species enhance the biofilm biomass, tolerance of the community to physical (*e.g*. desiccation), chemical (*e.g*. sodium dodecyl sulfate), and biological stress (*e.g*. antimicrobial agents), microbial virulence and the degradation of organic compounds and pollutants^13,14^. Based on these demonstrations, it was assumed that, the diversity of microbial interactions in complex ecosystems, such as soils, should affect biological functions at the ecosystem scale and that investigations of microbial network complexity might provide new insights into soil functioning and regulation^15^.

**Figure 1:**
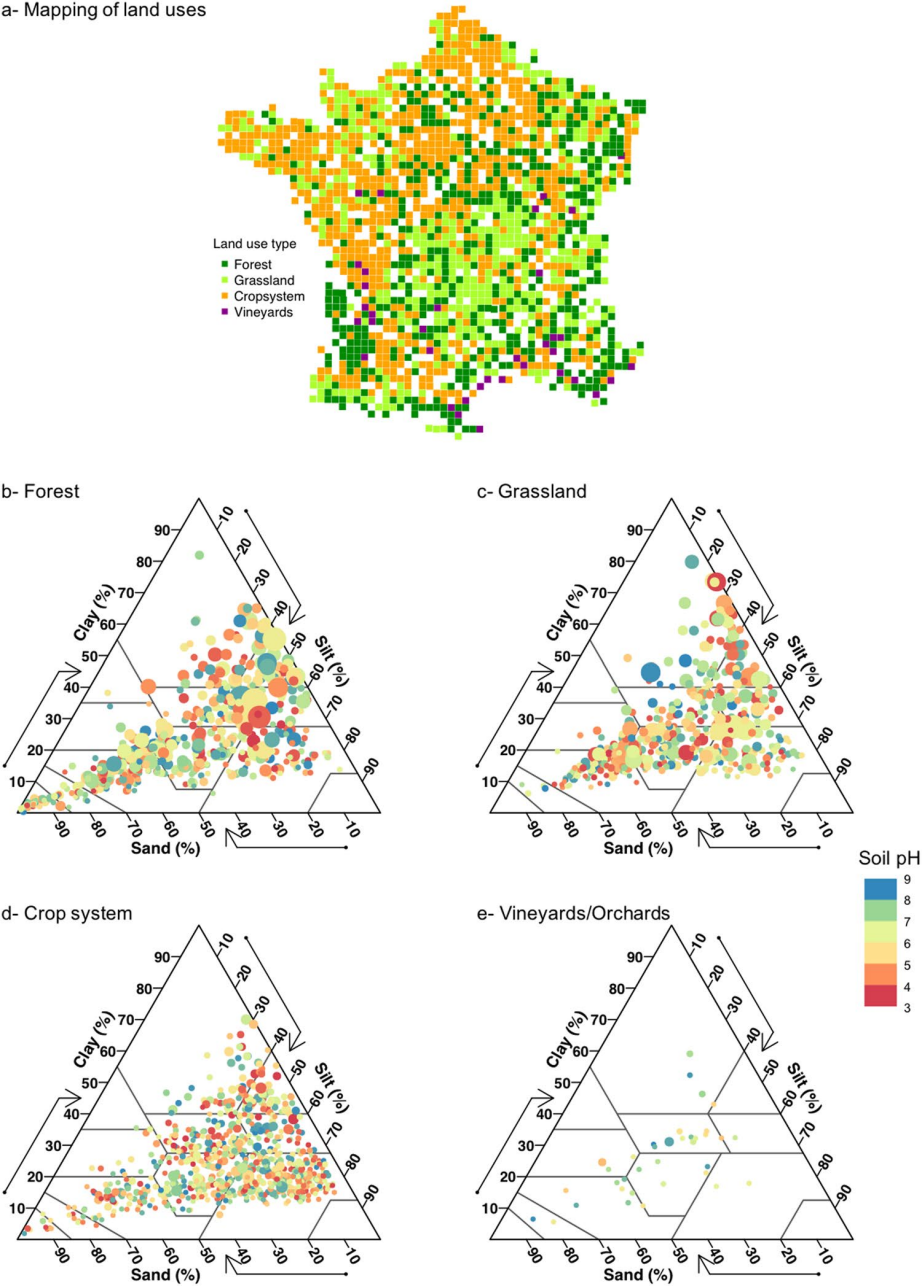
Characterization of soil samples: (**a**) Map of the 1717 sampling sites used to compute the network replicates and classification for the four land uses encountered in France. (**b**–**d**) Distribution of the RMQS soils in the USDA soil texture triangle for each land use respectively. Color legend from red to blue indicates soil pH and the circle size represents the relative amount of organic carbon in the soil.

Recently, soil bacterial co-occurrence networks have been shown to be impacted by soil physico-chemical characteristics^16,17^ and climate^18^, to vary with age of the plant cover^15^ and to change with the soil land use^19,20^. Both studies by Morriën and Lupatini demonstrated that the least complex bacterial networks were found in cropped soils, i.e. those most disturbed by agricultural practices, as compared to pastured or forest soils. These studies provided the first proof of the sensitivity of microbial interactions networks to land management but were only carried out on a local spatial scale. Now large-scale demonstrations are needed to determine the genericity of these conclusions^4^. In this study, we investigated soil bacterial co-occurrence networks on the scale of France by clustering soils according to the main land uses encountered across this country (Fig. 1a). Data were obtained from the French Soil Quality Monitoring Network (RMQS), which represents the most intensive soil sampling system on a wide spatial scale, due to its extensive area covered (5.5 10^5^ km^2^) and the high sampling resolution (about 2200 sites distributed along a systematic grid)^4^. The four land uses ranged from natural or semi-natural sites (forest and grassland) to agricultural sites (crops and vineyards), representing a gradient of cropping intensity, which integrated management of the plant cover and the degree of soil disturbance resulting from agricultural practices such as tillage, fertilization and pesticides inputs^21^ (see details in Material and Methods section). Previous studies based on RMQS data had revealed a lower microbial biomass but a higher bacterial taxonomic richness in vineyards and crop system than in forest and grassland soils^3,22^. Since these two microbial parameters were differently impacted by the land uses, investigating other microbial community parameters, such as the co-occurrence network, is a major concern to understand more comprehensively the response of microbial communities to the soil perturbations. Here, we evaluated and compared the structure of bacterial networks at the genus level by quantifying the complexity, the cohesion and the proportion of positive to negative relationships between land uses. We also identified the hub taxa for the bacterial network in each land use and related the ecological attributes of these taxa to the environmental and management context.

## Results

### Comparison of network structures between land uses

**Figure 2:**
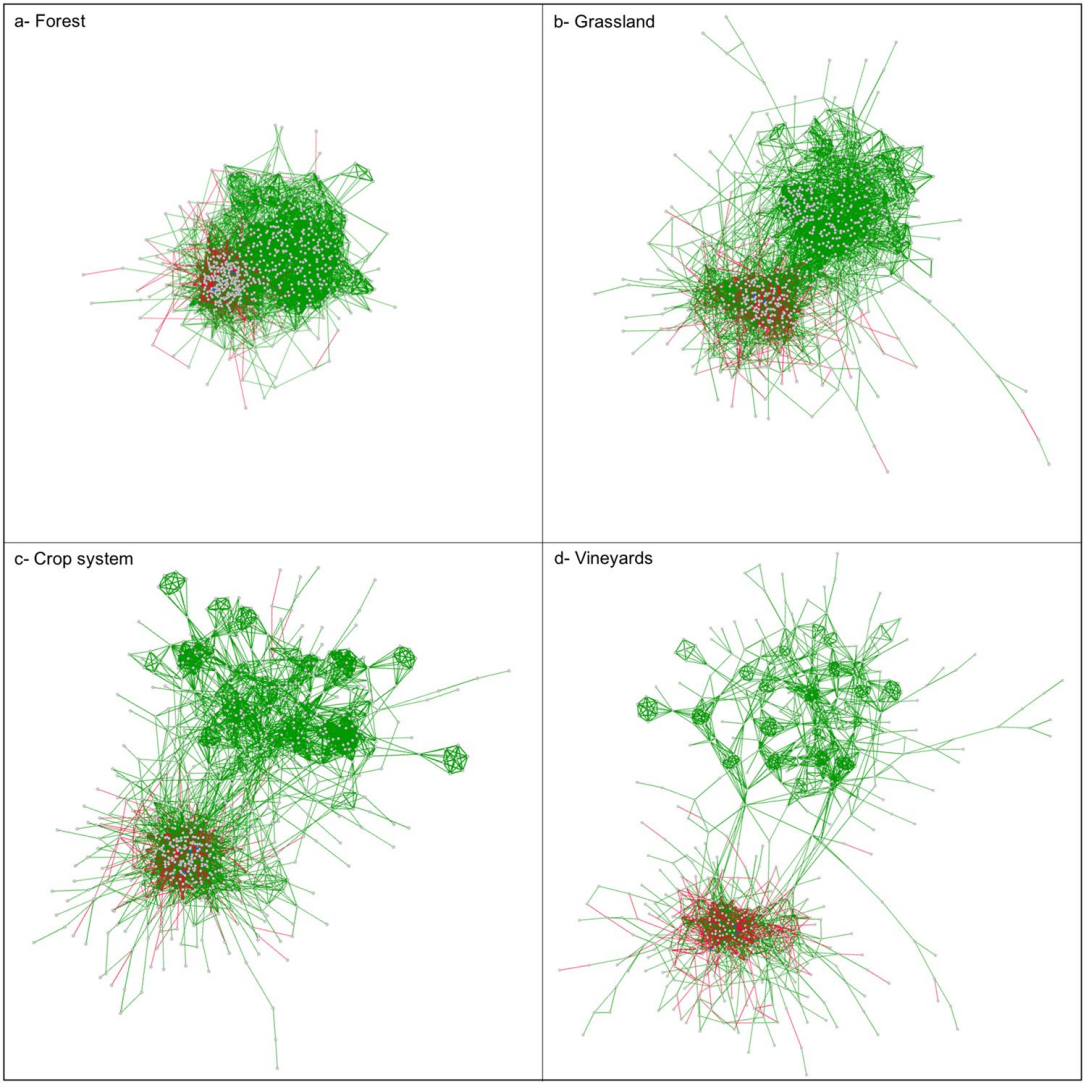
Visualization of the most complex network among the 100 replicates for the 4 types of land use. The red edges represent the negative links and the green edges represent the positive links. The most complex network was the one with the most links, the highest connectance and the highest average degree.

Graphically, the networks were composed of nodes and connections (the links). The connections were the significant correlations between the nodes, which corresponded to the genera occurring in the soils under the respective land use. The connection could be positive (green color) or negative (red color). The more the node was connected, the nearer it was to the heart of the network. Visual comparison of the networks for each land use revealed a significant shift in structure ranging from a highly connected, tightly closed structure for forests to a sparse, open structure for vineyards (Fig. 2). The bacterial networks in forest soils formed a dense cluster. In grassland soils, the cluster seemed to split into two parts with several long chains extending from the clusters. In soils under crop systems, one of clusters split into several large satellites which remained connected. In vineyards soils, many of the links seemed to be lost and the satellites were smaller and less inter-connected. Statistical comparisons of the network structures between the land uses confirmed a highly significant decreasing gradient in network complexity with forest > grassland > crop system > vineyards soils (Table 1). The number of links and the connectance were progressively reduced by 87% from forests to vineyards. Similarly, the average degree of the networks and the average path length decreased from forest to vineyards by 94% and 42%, respectively. In addition, the average number of links by genus was 16.2 in forest soils, 5.8 in grassland soils, 4.1 in crop system soils and only 2.0 in vineyards soils. A significant decrease in the number of positive and negative links was also observed from forest to vineyards (a loss of 85% and 92%, respectively). However, the greater decrease of negative links in vineyards, as compared to forests, led to a higher positive to negative ratio for vineyards. The positive to negative ratio increased from 2.9 in forest soils to 5.5 in vineyards soils.

**Table 1:** Community metrics for the 4 land uses: 6 indices of bacterial diversity and 7 indices of bacterial network. Different letters indicate a significant statistical difference between land uses, based on a parametric variance analysis followed by a Tukey HSD post-hoc comparison (n = 100 for each land use).

Community metrics		Forest			Grassland			Crop system			Vineyards		
		Mean	sd	Diff.	Mean	sd	Diff.	Mean	sd	Diff.	Mean	sd	Differences
*Diversity*	Richness in OTUs	1,083.3	224.0	c	1,238.1	157.1	b	1,305.9	167.9	a	1,342.8	152.3	a
	Shannon’s index for OTUs	5.27	0.39	c	5.41	0.32	b	5.50	0.31	a	5.57	0.23	a
	Eveness for OTUs	0.18	0.04	ns	0.18	0.04	ns	0.19	0.04	ns	0.19	0.03	ns
	Richness in *genera*	284.6	54.1	c	333.4	32.0	b	360.2	28.4	a	359.5	24.6	a
	Shannon’s index for *genera*	4.04	0.31	c	4.19	0.21	b	4.25	0.2	a	4.26	0.12	ab
	Eveness for *genera*	0.21	0.03	ns	0.2	0.03	ns	0.2	0.03	ns	0.2	0.02	ns
Co-occurrence network	Number of links	16,430.8	3,109.3	a	5,846.4	2,208.0	b	4,122.6	1,138.8	c	2,046.7	281.3	d
	Number of positive links	12,042.4	2174.9	a	4,539.5	1,917.4	b	3,101.2	697.3	c	1,794.0	172.2	d
	Number of negative links	4,388.4	1,123.6	a	1,306.8	486.5	b	1,021.4	475.8	c	342.7	115.3	d
	Positive:Negative links	2.9	0.7	c	3.7	1.8	b	3.5	1.2	b	5.5	1.7	a
	Connectance	0.008	0.002	a	0.003	0.001	b	0.002	0.0005	c	0.001	0.0001	d
	Average path length	0.139	0.007	a	0.129	0.019	b	0.116	0.016	c	0.080	0.009	d
	Average degree	16.2	3.1	a	5.8	2.2	b	4.1	1.1	c	1.0	0.3	d

Letters indicate differences of values between land uses. They are provided by Tukey-HSD pairwise comparison.

To investigate the assumption that network structure and complexity was directly related to community diversity, the richness, the Shannon index and the Shannon evenness in genera and in OTUs were also compared between land uses (Table 1). For both genus and OTU level, a significant increasing gradient (of about 25%, p-value < 0.001) was recorded along the cropping intensity gradient, the lowest richness occurring in forest soils (285 genera and 1083 OTUs on average) and the greatest richness in crop systems and especially in vineyards (360 genera and 1324 OTUs on average). The Shannon indices followed the same gradient as the richness, when the Shannon evenness’ did not differ significantly between land use.

### Identification of hub genera

The hub genera are those genera which exhibit the most links within a network, in other words the highest degree. Here, the hub genera were identified by statistically comparing the degrees obtained from the 100 replicates of network per land use, using a non-parametric pairwise test. The main hubs, their average degrees based on the 100 replicates and their rank within each land use are provided in Table 2. The quantitative analysis of these hubs highlighted a decrease of 80% in the degree (the number of links of the genera) from forests to vineyards. In forest, the primary hub was *Pirellula* with 337.7 links, whereas in grassland and crop systems, the primary hub was *Acidicaldus* with 183.9 links and 161.7 links, respectively, and *Bradyrhizobium* in vineyards with 73.9 links. *Pirellula* was also found among the 20 main hubs in grassland and vineyards soils whereas *Acidicaldus* was the third hub in vineyards and *Bradyrhizobium* was also among the 20 main hubs under crop systems. Interestingly, some of the major hubs were highly specific to a single type of land use, e.g. *Flavobacterium* found exclusively in forest soils and *Bradyrhizobium* found exclusively in cropped soils.

**Table 2:** Hub genera for the 4 land uses: the table summarizes the specific hubs for each land use and indicates the average degree of the genera for each of the land uses, the statistical difference with the other genera in the respective land use (given by a Kruskal-Wallis pairwise test) and, in parenthesis, the ranking of each genus among the most connected genera for the land use.

Top Hub Genera	Phylum-level classification of genus	Forest		Grassland		Crop system		Vineyards	
		Mean	Differences	Mean	Differences	Mean	Differences	Mean	Differences
*Flavobacterium*	*Bacteroidetes*	367.4	**ab (2)**						
*Blastochloris*	*Alphaproteobacteria*	361.0	abcd (4)	161.1	abcd (6)				
*Terrimonas*	*Bacteroidetes*	366.8	**abc (3)**	170.6	**ab (2)**	133.2	bc (4)		
*Pirellula*	*Planctomycetes*	377.7	**a (1)**	136.6	efg (14)			43.8	gh (11)
*Geothrix*	*Acidobacteria*	358.5	abcd (5)	155.0	bcde (7)	128.2	bcd (6)	42.6	gh (12)
*Stella*	*Alphaproteobacteria*	342.4	bcd (16)	170.2	**ab (3)**	151.0	**ab (2)**	63.9	abc (4)
*Acidicaldus*	*Alphaproteobacteria*			183.9	**a (1)**	161.7	**a (1)**	73.2	**ab (3)**
*Isosphaera*	*Planctomycetes*			162.7	abc (5)	149.9	**ab (3)**	53.7	cdef (7)
*Frigoribacterium*	*Actinobacteria*			144.7	cdefg (11)	128.9	bcd (5)	58.5	cde (6)
*Catenulispora*	*Actinobacteria*			126.2	g (18)	106.7	defg (16)	75.7	**a (2)**
*Bradyrhizobium*	*Alphaproteobacteria*					112.8	cdefg (12)	73.9	**a (1)**

Letters indicate differences of degree between nodes within each land use. They are provided by Pairwise Kruskal-Wallis Rank Test corrected by Bonferroni method.

## Discussion

### Global network structure changes between land uses

Visual analysis of the bacterial networks showed changes in the bacterial networks along the gradient of cropping intensity (Fig. 2). The soil bacterial networks seemed to progressively split and to shift from a single block with highly connected structure in forest soils to an open structure, full of small slightly inter-connected satellites in vineyards soils. This network structure in vineyards soils suggested an increasing isolation of several bacterial genera, which interacted little with the rest of the community. This particular evolution in network structure has not been described in previous studies of the impact of land uses or soil parameters. This might be explained by the lack of network visualization^19,20^ or the partial representation of networks^23,24^.

Regarding network metrics, the number of links and the connectance quantify the direct relationships and the network complexity. The average path length includes the indirect relationships, sometimes producing long chains with several intermediaries and translates the network cohesion. Finally, the positive to negative ratio indicates the balance between facilitative and inhibitive relationships within the network^11^. In our study, all these metrics revealed a significant shift in bacterial network structure along the gradient of cropping intensity. The progressive decrease in the number of links, the connectance and the average path length observed from forest to vineyards soils, demonstrated a loss of network complexity and cohesion as agricultural practices were intensified, and soils disturbed. Surprisingly, this result contrasted with the significant increase in bacterial richness observed across RMQS soils, where the sequence was: forests < grasslands < crop systems = vineyards^3^ (Table 1). Theoretically, the presence of more taxa within a community would imply more potential interactions. Despite the increased number of potential links, the most taxa-rich communities may not necessarily exhibit the most complex interaction networks^19^. This has already been observed in the case of soil disturbance. In Karimi *et al*., transplanting of samples from rural conditions to urban and industrial sites led to the loss of 40% of the links after 8 months, without any decrease of the microbial diversity^25^. Similarly, in another study^16^, the contamination of a forest soil used as a tailings dump decreased the number of links in the bacterial co-occurrence network by 25% compared to the pristine soil while the bacterial richness was increased by 20%. Interestingly, our results confirmed that the pool of taxa and the biotic relationships within the community responded independently to environmental perturbation^15^.

The observed decrease in network complexity and cohesion supports the hypothesis that cropping may enhance the isolation of bacterial taxa, as previously suggested by visual observation. Isolation can be enhanced in three ways. Firstly, it can result from the loss of microbial biomass and therefore of cells. This hypothesis has been supported by the decreasing microbial molecular biomass in crop systems and vineyards compared to forest and grassland^22^. If the total number of cells in soil is reduced, the probability of each cell encountering another and interacting, whether directly or indirectly, may be also reduced. Secondly, isolation can be caused by the stimulated metabolic independence of microorganisms in agricultural soils. Soil disturbance, produced by agricultural practices (such as tillage), is known to increase soil bacterial diversity and to preferentially stimulate some opportunistic and/or pathogenic populations^26^. These populations are self-sufficient and do not need to interact with others to be metabolically efficient and increase their fitness, which can therefore lead to even greater isolation of other bacterial populations. Finally, the isolation can be related to the weak spatial connectivity occurring between soil ecological niches in disturbed soils^27^. At the microscale, the structure of tilled soils is more homogeneous, and the pores are less connected than in soils under minimum or without tillage^28^. This can further induce the physical isolation of bacterial taxa in disturbed soils^29^. As microbial co-occurrence networks translate both the biotic interactions and niche-sharing^30^, the lower level of microbial networks cohesion (found in crop systems and vineyards) might be associated with the less spatially-structured distribution of ecological niches in such soils due to their frequent disturbance by tillage or fertilization. Altogether, these results suggest that forest and grassland soils represent a mosaic of connected ecological niches that are fully complete and shared by non-opportunistic bacterial genera when the soil structure and trophic resources are non-limiting^31,32^ (Fig. 2a,b). On the contrary, agricultural soils, especially vineyards, would consist of a mosaic of habitats with weakly-connected, partially-filled niches where the bacterial genera tend to be spatially and metabolically isolated by an altered soil structure and limited trophic resources^33,34^ (Fig. 2c,d). Unlike the other metrics, the ratio between positive and negative links increased from forest soils to vineyards soils. Although both positive and negative relationships decreased, the negative ones decreased more rapidly. This suggests that inhibition, rather than cooperation between bacteria taxa, was more affected by the cropping intensity. The weak availability of the soil organic matter in agricultural soils (Fig. 1) represented harsh conditions which could lead to selection of the most adapted taxa. Taxa with ecological attributes such as oligotrophy, ability to degrade recalcitrant matter or anaerobic metabolism, would be able to share this soil habitat^30,35^ and thus to maintain some of the positive relationships at the scale of bacterial network^30^. This supports the concept of resource-driven co-occurrence patterns^36^.

### Shift in hubs networks along the gradient of cropping intensity

The hubs represent the most connected taxa within a network^36,37^. Most hub genera identified in our study (Table 2) were consistent with a recent review of microbial keystone taxa in different ecosystems based on 37 studies published between 2011 and 2017^36^. *Rhizobiales* (like *Blastochloris* genus in our study) and *Terrimonas* genus have previously been recorded as hub/keystone taxa in forest and woodlands. In grasslands, bacteria belonging the phyla Bacteroidetes, Planctomycetes, Acidobacteria and Actinobacteria were also identified as hubs. And in agricultural soils, *Rhodospirillales* (*Stella* and *Acidicaldus* here), *Rhizobiales* (*Bradyrhizobium* here) and *Actinomycetales* (*Frigoribacterium* and *Catenulispora* here) were keystone taxa. Due to their high level of connectivity with other community members, these taxa could have a key role in the soil bacterial community of these land uses^36^.

Given the known ecological attributes of the bacterial hubs identified here, the progressive shift of these hubs along the gradient of cropping intensity might be partly due to the soil trophic level. In cropped soils (vineyards and crop systems), demonstrated to be the poorest of the RMQS soils in terms of organic carbon and nutrient contents^32^, the major contribution of *Bradyrhizobium*, *Acidicaldus* and *Isosphaera* to the network may be explained by their oligotrophic nature^38–40^. Rhizobia, such as *Bradyrhizobium*, are also known to develop highly specific symbioses with their host plant^41^, which might enhance their co-occurrence with other bacterial populations associated with the rhizosphere. Conversely, Bacteroidetes genera (*Flavobacterium* and *Terrimonas*) were the hubs of bacterial networks in forest and grassland soils, the richest of the RMQS soils in terms of organic matter content^32^. The bacterial phylum Bacteroidetes is known to be copiotrophic and more abundant in forest soils and therefore to interact strongly with other taxa^42,43^. However, few ecological data concerning the biotic interactions and ecological attributes of most of these genera are currently available, which therefore limits our ability to draw conclusions.

Interestingly, our results also revealed that *Flavobacterium* and *Terrimonas* were, at the same time, network hubs and two of the most abundant genera in forest soils^43^. Even if the most connected taxa might also be expected to be the most abundant, this hypothesis was not corroborated for the other land uses. The respective hub genera in grassland, crop system and vineyards soils had previously been shown to be only slightly abundant in these soils^43^. The identification of both abundant and hub taxa provides complementary information about the bacterial community ecology. It also reinforces the need to investigate different parameters describing soil microbial communities^11^ so as to better predict their responses to the changing environment and propose the most appropriate soil management practices.

### Robustness and limitation of the sampling and analytical strategies

This work is based on the most intensive, without *a priori*, soil sampling survey (about two thousand soil samples) focusing on a nation-wide scale. The sampling design covered the major environmental variability across a 550,000 km^2^ area^4^, thus precluding the bias associated with *a priori* samplings. This enable us to construct robust networks with a reasonable number of repetitions to verify our assumption concerning the effect of the cropping intensity. Compared to other studies based on 3 to 20 samples, our sampling design provides a cornerstone for robust analysis and conclusions about soil bacterial biogeography. The observed variability in network structure and metrics, (computed from 100 replicates) within each land use, was probably due to the variability in pedoclimatic conditions, i.e. the interacting soil characteristics, and/or agricultural practices recorded for each land use^4^. Nevertheless, according to the multi-dimensional plots (Fig. 1 and Supplementary Fig. 1), the environmental heterogeneity was similar for all four land uses. Moreover, as the local environment determined the microbial biomass^1^, the bacterial diversity^3^ and the distribution of taxa^43^, the network structure could also be influenced by the local spatial effect. In our sub sampling, the 30 sites covered an area ranging from 200,000 km^2^ to 550,000 km^2^ across a territory of 551,500 km^2^. However, the area covered had no impact on the network structure, whatever the land use (Supplementary Fig. 2 and Supplementary Table 1). The networks based on sites located closer together were not different from the others, which confirmed that the local environmental filters did not affect the bacterial network. We therefore assumed that the results obtained for the bacterial co-occurrence networks were only due to the different land uses and to the cropping intensities that they represent.

Our molecular analytical strategy is known to be highly robust^44^, although numerous biases inherent in amplicon library preparation such as DNA extraction^45^, amplification^46^, sequencing and inference of the patterns of organism abundance from library data pertaining to relative abundance, are also well-known^47^. Analyses were conducted in a consistent manner to remove errors due to sequencing and chimeras, and the datasets were rarefied to the same sampling depth (*i.e*. 10,000 reads per sample), so that relative changes in microbial taxonomic composition levels could be compared across samples, even if the biodiversity sampling was not exhaustive^47^. Nevertheless, the comparison of our results with those from other studies is mainly limited by our choice of 16S rRNA primers, which were designed to specifically target both bacteria and archaea diversity. The results obtained by using these primers were able to reveal taxonomic groups (for example *Holophaga*)^43^, rarely detected in previous soil studies. In addition to using these different primers, we also chose a finer but more time-consuming method of taxonomic assignment than the approach currently used in QIIME: Instead of assigning the seed sequence of each OTU, all reads in the dataset were individually assigned. This led to changes in relative abundance of the taxonomic groups within the community, probably in the actual structure of the co-occurrence network and finally the revelation of hub *genera*, probably underestimated in other studies.

## Conclusions

Altogether our study demonstrated that soil bacterial co-occurrence networks are different between land use types and are strongly shaped by the cropping intensity. We hypothesized that changes in the bacterial network would occur mainly in response to shifts in the heterogeneity and connectivity of the mosaic of microbial habitats as well as to the availability of C-substrates. Beyond the classical information obtained from bacterial richness or whole taxonomic composition, co-occurrence network analysis provides complementary insights into biotic interactions and niche connectivity, which could have repercussions on community stability^48^ and soil functioning^49^. Further investigations must now be focused on the dynamics of these networks to evaluate their response to environmental perturbations and also on the fungal community to obtain an overview of the soil microbial network.

## Material and Methods

### Sampling Design

Soil samples were obtained from the French Soil Quality Monitoring Network (“Réseau de Mesures de la Qualité des Sols”, RMQS) which is a soil monitoring network based on a 16 km regular grid across the 550 000 km^2^ French territory^1,3^. The soil profile, site environment, climatic factors, location, vegetation and land management for the 2173 RMQS monitoring sites have already been described. All samples were collected between 2002 and 2009. All sites have been geo-positioned with a precision of <0.5 m and the soil profile, site environment, climatic factors, vegetation and land use described^1^. In the middle of each 16 × 16 km square, 25 individual core samples were taken from the topsoil (0–30 cm) using an unaligned sampling design within an area of 20 × 20 m. These core samples were bulked to obtain a composite sample for each RMQS site. The soil samples were gently air-dried, sieved to 2 mm and then stored at −40 °C before analysis. Physico-chemical parameters were measured for each composite soil, *e.g*. particle-size distribution, pH water, organic C, N, C/N ratio, soluble P contents, calcareous, cation exchange capacity (CEC) or exchangeable cations (Ca, Mg). Physical and chemical analyses are available for 2,131 soils and were performed by the Soil Analysis Laboratory of INRA (Arras, France, http://www.lille.inra.fr/las). Available climatic data for the RMQS were monthly rainfall, evapotranspiration and temperature at each node of a 12 × 12 km² grid, averaged for the 1992–2004 period. These climatic data were obtained by interpolating observational data using the SAFRAN model. The RMQS site-specific data were linked to the climatic data by finding the closest node within the 12 × 12 km² climatic grid for each RMQS site. Land cover was recorded according to the coarse level of the CORINE Land Cover classification (IFEN, http://www.statistiques.developpement-durable.gouv.fr/donnees-ligne/li/2539/0/base-donnees-geographique-corine-land-cover-clc.html), which consists of a rough descriptive classification into five classes: forest, croplands, grasslands, perennial crops (corresponding to vineyards and orchards) and others. All these data were available in the DONESOL database^1,32^.

### Characterization of the gradient of cropping intensity

The gradient of cropping intensity was composed of four land uses that were characterized by the management and turnover of the plant cover, the duration of the management plan, and the frequency and intensity of interventions which disturbed the soil: (i) The “forest” land use grouped together broad-leaved forests, coniferous forests, poplar grove and mixed forests, which could be natural or planted. The management plans were often established over 10 to 150 years with few perturbations until the wood cutting. These forest soils are not tilled, and few engines pass over them. There is no fertilization and no pesticide input. Turnover of the plant cover is on the scale of decades. (ii) The “grassland” land use grouped together 6 to 10-years old seeded grassland, >10-years seeded or natural grassland, unproductive permanent grasslands and meadows. Some of these grasslands may be seasonally pastured. Tillage is rare. In France, less than 30% of grasslands receive organic manure and 40% on average receive low doses of mineral fertilizer (50 kgN/ha on average). Only 6% of grasslands receive pesticides (data from the Ministry of Agriculture, 2011). Turnover of the plant cover exceeds 6 years. (iii) The “crop system” land use grouped together annual crops in monoculture, annual crops associated with permanent crops, complex cultivation patterns, market gardening and ornamental horticulture, or fallow land. These systems are strongly managed to ensure satisfactory yields. During the year, the different interventions concern soil tillage, seedling, fertilization and pesticides applications. Even if the frequency and intensity of interventions can vary considerably across the crop systems, they take place every year. Turnover of the plant cover is annual. (iv) The “vineyards” land use grouped together vineyards, orchards and others perennial tree crops. Like the crop systems, the annual interventions concern soil tillage, fertilization and pesticides applications. However, the frequency and intensity of pesticides input are greater than for annual crop systems due to the perennial implantation of the vines and trees.

Overall, the forest soils are the least disturbed and the vineyards soils are the most disturbed by the agricultural practice and interventions over time. These agricultural practices have also affected the soil nutrient status by determining the amount and the quality of organic matter. Thus, these land uses ranged from natural or semi-natural sites (forest and grassland) to agricultural sites (crop and vineyards). All soil samples were therefore classified according to the land use of the site: 492 forest soil samples, 464 grassland soil samples, 740 cropped soil samples and 36 vineyard or orchard soil samples.

### Molecular characterization of bacterial communities

The analysis of these 2173 samples required the rigorous standardization of the range of different molecular tools involved in soil DNA extraction and sequencing technology. The following protocols were applied.

#### Soil DNA extraction and purification

Microbial DNA was extracted and purified from 1 g of each of the 2173 composite soils (composed of a bulk of 25 individual core soils) sampled at each RMQS site, using the previously-described GnS-GII procedure^45^. Crude DNA extracts were quantified by agarose gel electrophoresis stained with ethidium bromide, using calf thymus DNA as standard curve^1^. Crude DNA was then purified using a MinElute gel extraction kit (Qiagen, France) and quantified using a QuantiFluor staining kit (Promega, USA), prior to further investigations.

#### PCR amplification and pyrosequencing of 16S rRNA gene sequences

A 16S rRNA gene fragment targeting the V3-V4 regions to characterize bacterial diversity was amplified using the primers F479 (5′-CAGCMGCYGCNGTAANAC-3′) and R888 (5′-CCGYCAATTCMTTTRAGT-3′) with the method described previously^45^. From the 2173 DNA soil samples, 2132 soil samples were successfully amplified. The PCR products were then purified using a MinElute PCR purification kit (Qiagen) and quantified using the QuantiFluor staining kit (Promega, USA). A second PCR of 7 cycles was then run twice for each sample under similar PCR conditions, with purified PCR products as matrix (7.5 ng of DNA were used for a 25 µl mix of PCR) and dedicated fusion primers (‘F479/AdaptorB’, ‘R888/MID/AdaptorA’) integrating the required adaptors, keys and multiplex identifiers at the 5′ extremities. All duplicated PCR products were then pooled, purified using a MinElute PCR purification kit (Qiagen), and quantified using the QuantiFluor staining kit (Promega, USA). For all libraries, equal amounts from 30 samples were pooled, and then cleaned to remove excess nucleotides, salts and enzymes using the Agencourt AMPure XP system (Beckman Coulter Genomics). 100 µl of TE buffer (Roche) was used for the elution. Pyrosequencing was then carried out on a GS FLX Titanium (Roche 454 Sequencing System) by Genoscope (Evry, France).

#### Bioinformatics sequence analysis

Bioinformatic analyses were done using the GnS-PIPE pipeline developed by the GenoSol platform (INRA, Dijon, France) (availability: 10.5281/zenodo.1123425) and previously detailed^3,43^. After sequencing, 49,794,516 raw reads were obtained for the 2132 soil samples. After the different preprocessing and filtering steps (detailed previously^3^), 32 634 692 high-quality reads (range of sequencing depth: 48 reads to 49 926 reads by sample) were kept. The number of high-quality reads for each sample was then “rarefied” (i.e. 10 000 high-quality reads for each sample) by random selection to allow efficient comparison of the datasets and avoid biased community comparisons and rarefaction curves. Thus, 1798 soil samples were kept for subsequent analyses, encompassing a total of 17 980 000 reads. A final post-processing step was then applied to this global dataset, as already described regarding for microbial richness across France^3^, to filter the potentially artefactual reads. Briefly, the 17 980 000 reads from all samples were firstly aligned and clustered at 95% of similarity into OTUs. Thereafter, all OTUs that occurred only once in the overall dataset and contained only a single read, were removed. This post-processing step reduced the number of total OTUs from 184 812 to 83 917 (more than 50% lost), but the number of reads only from 17 980 000 to 17 866 981 (less than 1% lost). The number of deleted reads by sample was 62 ± 60 on average (minimum: 10, maximum: 1093). Finally, all kept reads were then compared with the dedicated reference database derived from SILVA to independently determine the composition of each soil community at the *genus* level (procedure, database and programs available online: 10.5281/zenodo.1065438 and 10.5281/zenodo.1064170). The final dataset was composed of 1798 samples with 10 000 sequences per sample distributed among 1355 genera^43^. Unknown sequences represented an average of 11% by sample at the *genus* level. All raw data sets are publicly available in the EBI database system (in the Short Read Archive) under project accession no PRJEB21351.

### Statistical and co-occurrence network analyses

Comparing the bacterial networks between the four land uses required: i) standardization of the number of soils used to compute the network by land use to avoid a sampling size effect and ii) network replicates which integrated the natural heterogeneity of the soils within each land use. As vineyards provided only 36 soil samples on the territory scale, we standardized the sampling size at 30 sites per network for all land uses, drawn from the respective pool of soils. Thus, the minimum number of combinations (1 947 792) ensured that each network was computed from a unique combination of sites. The number of replicates used to evaluate the range of variation within and between land uses was set at 100. The 30 samples were drawn independently for each of the 100 network replicates. Numerical sampling of the sites was monitored. Among the 1732 sites classified as forest, grassland, crop system or vineyards, 1717 were sampled at least once. This replication procedure ensures that more than 99% of the available sites were sampled, standardized analysis of all four land uses, while being representative of French soils in general.

Then, for each replicate, network computation was based on a contingency matrix which provided the relative abundances of 1355 bacterial genera for the 30 randomly-selected soil samples. The Spearman correlation coefficient for each pair of genera was used as a similarity index to estimate taxa co-occurrence^50^. A correlation was considered as robust and non-random if the p-value was below 0.05 after correction using the False-Discovery Rate method^51^. By choosing this threshold, only correlations with a coefficient above 0.50 were kept. The positive and negative correlations were respectively interpreted as co-occurrence (facilitation) and co-exclusion (antagonistic) relationships. To describe the topology of the resulting networks, a set of metrics were calculated, namely the number of links, the number of positive links, the number of negative links, the ratio between the positive and negative links, the connectance defined as the proportion of potential links which are significantly observed, the average path length and the average degree (for more details, see^11^). Due to the homoscedasticity between land-use and the Gaussian distribution of the model residuals, these metrics were then compared between the four land uses by applying a parametric analysis of variance and a Tukey HSD post-hoc comparison. The degree (defined as the number of edges of each node) of the 20 most connected genera was recorded for all 100 replicates of all four land uses and then statistically compared within each land use by applying a pairwise Kruskal-Wallis rank test corrected by the Bonferroni method. The genera with the highest average degree were then considered as hub genera.

In addition, the bacterial diversity was evaluated for each land use at the taxonomic level of OTUs and genera. Three indices were computed: a- the richness *N*_0_ which corresponds to the number of taxa, b- the Shannon entropy index *H* = −*Σ*_*i*_ *(P*_i_*log P_*i*_), where P_i_ is the probability of finding the taxon i and c- the Shannon eveness *E* = *exp(H)/N*_0_. These indices were compared between the four land uses by applying a parametric analysis of variance and a Tukey HSD post-hoc comparison.

## Supplementary information

Supplementary Figures and Table

## Data Availability

All raw data sets are publicly available in the EBI database system (in the Short Read Archive) under project accession no PRJEB21351.
