# Supplementary material for: Biogeography of Soil Bacterial Networks along a Gradient of Cropping Intensity
Source: Sci Rep. 2019 Mar 7;9:3812. doi: 10.1038/s41598-019-40422-y (PMC6405751; doi:10.1038/s41598-019-40422-y)
Supplement: Supplementary file 1 — Supplementary Figures and Table [file 41598_2019_40422_MOESM1_ESM.pdf]

Supplementary information

Biogeography of Soil Bacterial Networks along a Gradient of Cropping Intensity

Battle KARIMI[1], Samuel DEQUIEDT[1], Sébastien TERRAT[1], Claudy JOLIVET[2], Dominique ARROUAYS[2], Patrick WINCKER[3], Corinne CRUAUD[3], Antonio BISPO[2,4], Nicolas CHEMIDLIN-PREVOST BOURE[1], Lionel RANJARD[1]*.

[1] *Agroécologie, AgroSup Dijon, INRA, Université Bourgogne Franche-Comté, F-21000 Dijon, France*

[2] *INRA Orléans - US 1106, Unité INFOSOL, Orléans - France*

[3] *CEA / Institut de Génomique / Genoscope, Evry cedex - France*

[4] *ADEME, Service Agriculture et Forêt, Angers, France*

Supplementary Figure 1: PCA on all the environmental variables measured on the 1732 sites.

Supplementary Figure 2: Spatial effect on bacterial co-occurrence networks.

Supplementary Table 1: Spatial effect on bacterial co-occurrence networks (statistical test).

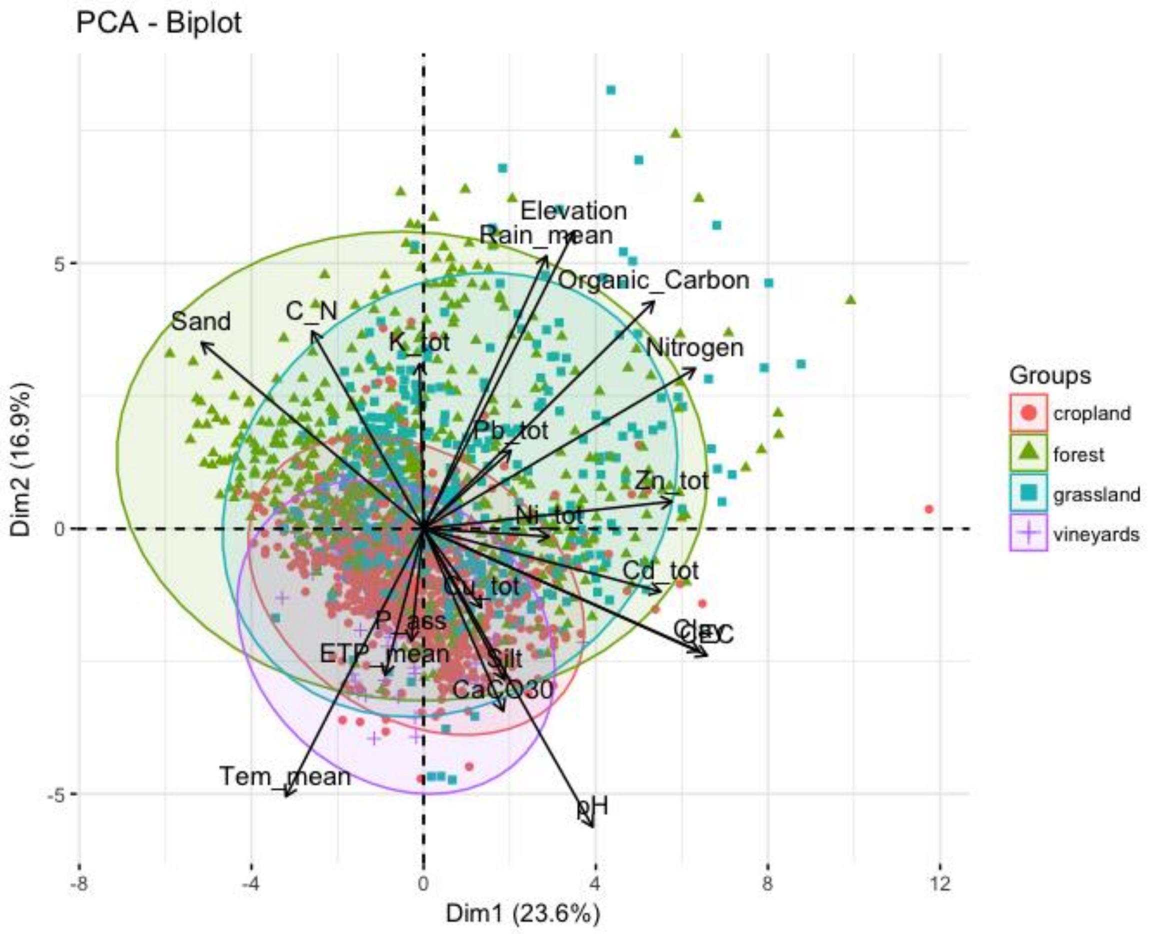

Supplementary Figure 1: PCA on all the environmental variables measured on the 1732 sites, *i.e.* soil parameters, climate factors and elevation. The ellipses represent the distribution of the sites for each land use (95% confidence interval).

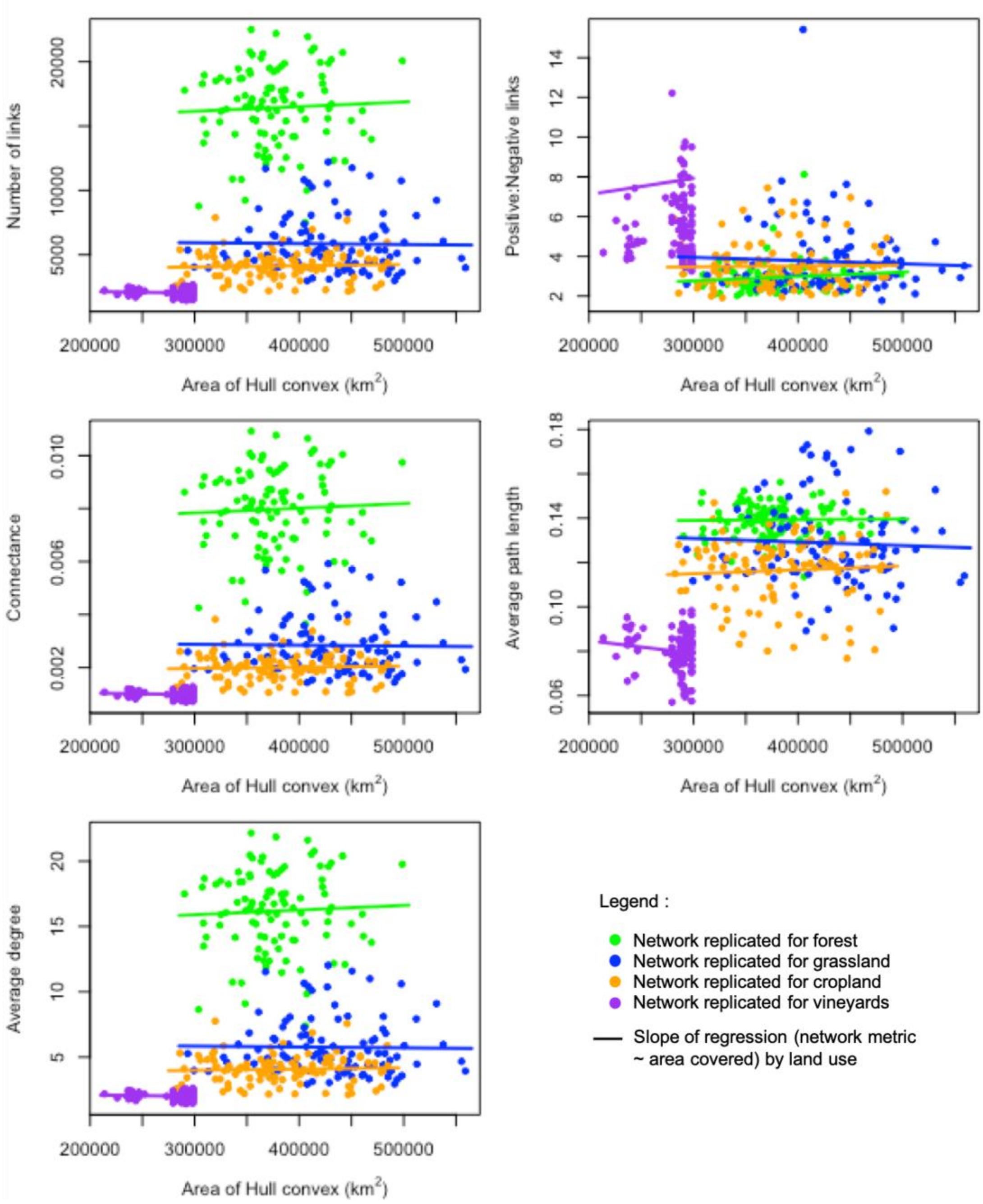

Supplementary Figure 2 : Spatial effect on bacterial co-occurrence networks. To ensure that the local effects did not impact the co-occurrence analysis, we calculated the area covered by the sub-sampling (area of the Hull convex based on the spatial coordinates of the 30 random sites) for the 100 repetitions of network and we correlated this area to the 5 main network metrics. Whatever the network metric and the land use, the area covered by the sub sampling had not significant effect on the value of the metric (slopes were not significant, see the Supplementary Table 1). An interesting point was that all the intercepts were significantly different from 0 (pv $< 10^{-2}$), which means that the average value was not random. We can note that the range of area for vineyards was lower than for other land uses. This was due to a distribution of vineyards sites in some French regions as shown on the figure 1. Despite this more localized distribution, the distance between the sites kept a non-significant effect on the co-occurrence network.

Supplementary Table 1: Spatial effect on bacterial co-occurrence networks. Effect of covered area was tested on 5 metrics of bacterial co-occurrence network. All the intercepts were significantly different of 0 and all the slopes were not significant according a linear regression.

Network variable	Spatial variable	Land use	Intercept	p-value	slope	p-value
Number of links	Area of Hull convex	Forest	15070	1.83E-06	3.60E-03	0.65
P:N ratio	Area of Hull convex	Forest	2.106	3.59E-03	2.02E-06	0.28
Connectance	Area of Hull convex	Forest	0.007	1.83E-06	1.75E-09	0.65
Average Path length	Area of Hull convex	Forest	0.138	<2E-16	3.25E-09	0.86
Average degree	Area of Hull convex	Forest	14.840	1.83E-06	3.54E-06	0.65
Number of links	Area of Hull convex	Grassland	6136	7.12E-04	-6.77E-04	0.87
P:N ratio	Area of Hull convex	Grassland	4.436	1.92E-03	-1.63E-06	0.61
Connectance	Area of Hull convex	Grassland	0.003	7.12E-04	-3.28E-10	0.87
Average Path length	Area of Hull convex	Grassland	0.1356	3.86E-14	-1.59E-08	0.66
Average degree	Area of Hull convex	Grassland	6.043	7.12E-04	-6.66E-07	0.87
Number of links	Area of Hull convex	Cropland	3763	1.65E-05	9.43E-04	0.66
P:N ratio	Area of Hull convex	Cropland	3.345	2.49E+04	4.11E-07	0.86
Connectance	Area of Hull convex	Cropland	0.002	1.65E-05	4.57E-10	0.66
Average Path length	Area of Hull convex	Cropland	0.110	3.68E-15	1.75E-08	0.57
Average degree	Area of Hull convex	Cropland	3.705	1.65E-05	9.28E-07	0.66
Number of links	Area of Hull convex	Vineyards	2361	5.16E-09	-1.12E-03	0.40
P:N ratio	Area of Hull convex	Vineyards	5.479	<2E-16	8.15E-06	0.29
Connectance	Area of Hull convex	Vineyards	0.001	5.16E-09	-5.41E-10	0.40
Average Path length	Area of Hull convex	Vineyards	0.096	7.52E-13	-5.81E-08	0.16
Average degree	Area of Hull convex	Vineyards	2.324	5.16E-09	-1.10E-06	0.40
